# The Influence of *Pichia kluyveri* Addition on the Aroma Profile of a Kombucha Tea Fermentation

**DOI:** 10.3390/foods12101938

**Published:** 2023-05-10

**Authors:** Niël van Wyk, Julia Binder, Marie Ludszuweit, Sarah Köhler, Silvia Brezina, Heike Semmler, Isak S. Pretorius, Doris Rauhut, Martin Senz, Christian von Wallbrunn

**Affiliations:** 1 Department of Microbiology and Biochemistry, Hochschule Geisenheim University, 65366 Geisenheim, Germany; niel.wyk@hs-gm.de (N.v.W.); julia.binder@mail.hs-gm.de (J.B.); silvia.brezina@hs-gm.de (S.B.); heike.semmler@hs-gm.de (H.S.); doris.rauhut@hs-gm.de (D.R.); 2 ARC Centre of Excellence in Synthetic Biology, Department of Molecular Sciences, Macquarie University, Sydney, NSW 2109, Australia; 3 Department Bioprocess Engineering and Applied Microbiology, Research and Teaching Institute for Brewing in Berlin, Seestraße 13, 13353 Berlin, Germany; m.ludszuweit@vlb-berlin.org (M.L.); sa-koehler@web.de (S.K.); m.senz@vlb-berlin.org (M.S.)

**Keywords:** kombucha, acetate esters, *Pichia kluyveri*, flavor, sensory analysis, total phenolics

## Abstract

Traditional kombucha is a functional tea-based drink that has gained attention as a low or non-alcoholic beverage. The fermentation is conducted by a community of different microorganisms, collectively called SCOBY (Symbiotic Culture of Bacteria and Yeast) and typically consists of different acetic acid bacteria and fermenting yeast, and in some cases lactic acid bacteria that would convert the sugars into organic acids—mostly acetic acid. In this study, the effect of including a *Pichia kluyveri* starter culture in a kombucha fermentation was investigated. *P. kluyveri* additions led to a quicker accumulation of acetic acid along with the production of several acetate esters including isoamyl acetate and 2-phenethyl acetate. A subsequent tasting also noted a significant increase in the fruitiness of the kombucha. The significant contribution to the aroma content shows the promise of this yeast in future microbial formulations for kombucha fermentations.

## 1. Introduction

Kombucha is a traditional Chinese tea-based drink that has gained significant popularity with consumers across the world over the past decade [1]. The global kombucha tea market has also shown enormous growth in recent years and will continue to grow healthily over the next five years with a projected compound annual growth rate of 24.8%, which corresponds to a value of USD 10.26 billion in 2028 [2]. Traditional kombucha combines a unique sweetness and sourness along with the expected tea notes and is considered a functional beverage. This implies that kombucha has potential health benefits, though these claims are yet to be substantiated with clinical data [3,4,5]. Kombucha has also garnered attention due to its low or no alcohol content which has become an important consideration among a growing number of consumers [6,7,8]. In addition to its industrial-scale fermentations, kombucha is also a popular homemade drink, with many households experimenting with alternative substrates other than tea [9]. 

The starting substrate of a traditional kombucha fermentation consists of a black or green tea that is sweetened (mostly with sucrose) [10]. The microflora responsible for the fermentation is usually obtained from a previous fermentation and consists of the microorganisms found in the cellulose-rich pellicle as well as the liquid (or soup) part of the kombucha. This microflora is collectively called by the acronym SCOBY, which stands for ‘symbiotic culture of bacteria and yeast’ and consists of a core microflora of yeast, acetic acid bacteria and sometimes lactic acid bacteria. Yeasts often isolated from kombucha include, but are not limited to, members from the genera, *Brettanomyces*, *Candida*, *Saccharomyces*, *Schizosaccharomyces* and *Zygosaccharomyces* [11,12]. The acetic acid bacteria include members from *Acetobacter*, *Gluconobacter* and *Komagataeibacter* [13] whereas *Lactococcus* spp. and *Lactobacillus* spp. represent the main lactic acid bacteria isolates [14]. SCOBY drives the main fermentation process, which is to convert the sugars to organic acids. First, the yeast converts the sugars to ethanol, whereafter the bacteria (especially the acetic acid bacteria) oxidize the ethanol to acetaldehyde and then to acetic acid. Minor acids, such as gluconic acid, glucuronic acid and lactic acid, might also be produced during the fermentation while tea polyphenols might also be modified [15]. 

It is quite clear that the microbial composition of SCOBY plays an all-encompassing role in all outcomes of a kombucha fermentation. Researchers have started to investigate the effect of adding both bacterial and yeast starter cultures to a kombucha fermentation which has shown marked differences in the antioxidant and aroma content of the beverage [16,17]. 

The film-forming yeast *Pichia kluyveri* has been used as a starter culture for the production of many foodstuffs including wine [18,19,20], chocolate [21,22] and beer [23,24]. *P. kluyveri* was shown to possess remarkable alcohol acetyltransferase activity as its addition to a fermentation led to a dramatic increase in acetate esters levels. These esters include ethyl acetate (imparting a nail polish odor), isoamyl acetate (banana), 2-phenethyl acetate (roses), 3-sulfanylhexyl acetate (passion fruit) and several terpene acetates, all of which are important aroma determinants in beverages [25,26,27]. 

From a microbial point of view, it can be said that the kombucha matrix and the traditional manufacturing process do not represent optimal growth conditions for the microorganisms contained. Due to factors such as oxygen limitation, low pH values, nutrient gradients and tea phytochemicals, traditional kombucha fermentations can last between one and three weeks and thus complicates the standardization of a kombucha fermentation. Therefore, possibilities to accelerate the process without sacrificing product quality are of interest. In this context, the present study investigated the impact of adding a *P. kluyveri* starter culture, a yeast not commonly found as part of a SCOBY, to a traditional kombucha fermentation.

## 2. Materials and Methods

### 2.1. Fermentation Set-Up

The SCOBY used in the study comprised a pellicle from a previous kombucha tea fermentation of roughly equal size, as well as the liquid part (called the ‘soup’) of the ferment. The soup from a previous fermentation comprised 10% of the volume of the new fermentation. The starting material for the kombucha fermentation consisted of boiled water, 2 g/L black tea (Echte Ostfriesische Mischung, Broken Silber, Thiele Tee, Emden, Germany) and 50 g/L food-grade sucrose (Südzucker, Mannheim, Germany). The sucrose was dissolved in reverse-osmosis water and the black tea was added in a tea strainer after approximately 5 min of infusion. The infusion was cooled down to room temperature prior to sucrose addition. A commercial *Pichia kluyveri* strain, VINIFLORA^®^ FROOTZEN^®^ (Chr. Hansen Holding A/S, Hørsholm, Denmark) was used in the study. A 50 mL preculture was grown overnight in liquid YEPD (10 g/L yeast extract, 20 g/L glucose, and 20 g/L peptone) whereafter the cells were collected via centrifugation, washed with phosphate-buffered saline (PBS) and resuspended in an appropriate amount of the tea–sucrose mixture. It was then inoculated into the sweetened tea–SCOBY mixture at a cell concentration of approximately 1 × 10^7^ cells/mL. The kombucha fermentations were carried out in 1.7 L beakers covered with sterilized linen cloth. *P. kluyveri* was also inoculated into the sweetened-tea substrate without the addition of a SCOBY. Fermentations were incubated at room temperature without shaking. Samples were taken daily for HPLC analysis, while spectrophotometric analysis was conducted on Day 0 and Day 4, GC–MS analysis was carried out on Day 4.

### 2.2. Sensory Analysis

After the fourth day of the fermentation, kombucha samples were pasteurized by placing the samples in a water bath at 80 °C for 5 min, whereafter the samples were cooled and filtered through a coffee filter. Samples were stored for 4 days at 4 °C and brought to room temperature shortly before sensory evaluation. For this purpose samples were tasted in 80 mL scale in one session and characterized using an already established descriptive analysis scheme for sour fermented beverages in a non-blinded manner [28]. The panel of six consisted of two trained sensory experts and four tasters with relevant experience in tasting sour fermented beverages with an average age of 31.7 ± 6.3 and a male to female ratio of 1:2. 

### 2.3. Analysis of Kombucha Samples 

Standard operating procedures established by the analysis team at the Department of Microbiology and Biochemistry and the Department of Beverage Technology at Geisenheim University were followed in order to analyze the composition of the Kombucha fermentations. 

#### 2.3.1. High-Performance Liquid Chromatography (HPLC)

The concentrations of the organic acids, ethanol as well as the sugars fructose, glucose and sucrose of the samples taken daily were determined with HPLC, based on a method previously described [29]. 

#### 2.3.2. Gas Chromatography–Mass Spectrometry (GC–MS)

The concentrations of the major acetate esters of samples taken on Day 4 were determined using headspace gas chromatography coupled with mass spectrometry as outlined previously [30]. 

#### 2.3.3. Phenolic Content Determination

The Folin–Ciocalteu method was used with 50 mL of the samples taken on Day 0 and Day 4 as described previously [31]. A photometric automatic analyzer ARENA 20XT, (Thermo Scientific, Waltham, MA, USA) and the software Arena V.7.2AR1 was used.

### 2.4. Statistical Analyses

Fermentations were conducted in triplicate. A one-way analysis of variance followed by a Tukey post-test was used to compare the aroma compound concentrations of the different treatments. Analysis was performed using GraphPad-Prism^®^ Version 5.03 (GraphPad Software, San Diego, CA, USA). Significant differences between samples for each sensory attribute were determined via un-paired Student’s t-test in Microsoft Excel 2013 (Microsoft Cooperation, Redmond, WA, USA).

## 3. Results and Discussion

### 3.1. Fermentation Parameters

A traditional black tea-based kombucha fermentation was conducted and the effect of adding a *P. kluyveri* starter culture to the fermentation was investigated. 

**Figure 1:**
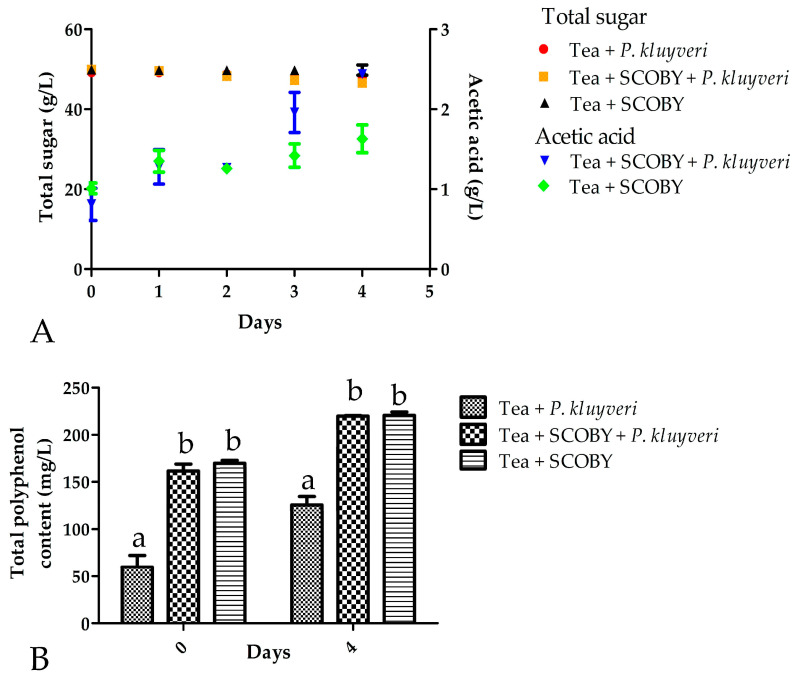
Influence of a *Pichia kluyveri* starter culture on the total sugar, acetic acid content (**A**) and total phenolic content (**B**) of the kombucha fermentation as determined via HPLC and spectrophotometric methods. Values are the average of three replicates. The error bars indicate the standard deviation.

Figure 1A illustrates the accumulation of acetic acid during the first four days of fermentation. While little sugar has been used during this period in any of the samples, we observed a clear increase in acetic acid levels with the added *P. kluyveri* after three days of incubation, which reached approximately 2.5 g/L after four days. This is in concurrence with previous reports that *P. kluyveri* additions to a fermentation result in higher levels of acetic acid albeit in other food matrices, such as grape must and cacao bean [18,21]. We did not observe gluconic or glucuronic acid in any of our samples.

No ethanol concentration above 0.5% *v*/*v* could be measured (results not shown) which could allude to the strong microbial activity of the acetic acid bacteria within the SCOBY that would consume all available ethanol whilst only producing acetic acid. The fermentations without any SCOBY (i.e., the tea + *P. kluyveri* fermentations) displayed little activity during the four-day-long fermentation as no acetic acid could be detected, which confirms the negligible growth that *P. kluyveri* has on sucrose as a carbohydrate substrate [32]. *P. kluyveri* thus relies on the invertase activity of SCOBY to utilize the monomers glucose and fructose as carbohydrate source.

Figure 1B shows the difference in total phenolic content from the onset of the fermentation until Day 4. There was no difference in the phenolic content with the addition of *P. kluyveri,* implying it has a minimal impact on these components, although closer inspection on the specific species of phenolic components is warranted to rule out any changes to the profile of the phenolic components. The fermentations without any SCOBY also showed a similar increase in phenolic content after four days and could suggest that the increase is not microbiologically induced. The increase in phenolic content is consistent with a kombucha fermentation as the increase in acid content and the enzymatic action of the members of the SCOBY lead to the release of more phenolic compounds [33,34].

### 3.2. Aroma Components from the Kombucha Fermentation

Table 1 shows the acetate ester content measured in the kombucha fermentations after four days. Three acetate esters (ethyl acetate, isoamyl acetate, and 2-methylbutyl acetate) were only detected in the samples where *P. kluyveri* was added whereas the 2-phenethyl acetate levels were 47 times higher than the fermentations without *P. kluyveri*. Interestingly, the fermentations without a SCOBY also produced 2-phenethyl acetate which could be ascribed to the trace amounts of glucose and fructose present in the food-grade sucrose, or a certain proportion that hydrolyzes into the monomers under the acidic conditions, that allows for limited amount of growth. Apart from the ethyl acetate, all other acetate esters far exceeded their respective odor threshold range.

### 3.3. Sensorial Analysis

**Figure 2:**
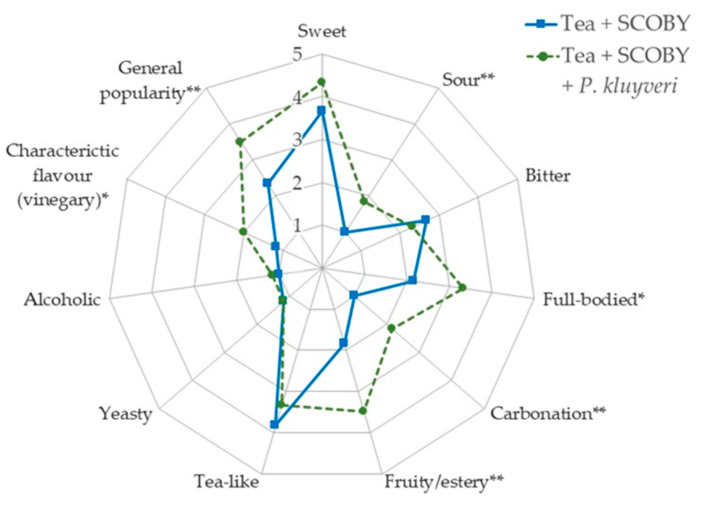
Organoleptic evaluation of the fermented products to assess the influence of the addition of the *Pichia kluyveri* starter culture. Blue line is the kombucha fermentation without the addition of *P. kluyveri* and the green dashed line is with the addition of *P. kluyveri*. The rating scale of the descriptive analysis ranges from 1 (imperceptible) to 5 (very pronounced). The rated general popularity of the taste ranges from 5 (very good) to 1 (bad taste). Significant differences between samples for each attribute were determined via unpaired Student’s *t*-test and are marked with asterisks (* *p* < 0.05, ** *p* < 0.005).

A small-scale sensorial analysis was conducted on the kombucha prepared with and without the addition of a *P. kluyveri* starter culture. In general, the addition of the *Pichia* starter culture led to accelerated beverage fermentation. This can be seen from the decrease in the attributes ‘bitter’ and ‘tea-like’ and the increase in the attributes ‘sour’, ‘full-bodied’, ‘carbonation’, ‘fruity/ester’, ‘characteristic flavour’ and generally better rating (Figure 2). Of note was the expected increase in the fruitiness of the kombucha with the *P. kluyveri* addition. From the view of industrial production and quality assurance aspects, the addition of *P. kluyveri* could be an interesting possibility to better control and accelerate the fermentation process.

## 4. Conclusions

This work shows the remarkable impact that *P. kluyveri* addition has on the aroma composition of a traditional kombucha fermentation. Its proven acetate ester production capability could thus be transferred to fermentation settings where it is not commonly found and could ultimately be used as a tool to direct the aroma profile and accelerate the kombucha fermentation process. The advantage of using *P. kluyveri* is that it provides a strong “fruity” flavor without the use of artificial flavorings.

## Figures and Tables

**Table 1:** Acetate ester content of the kombucha products. Values are the average of three replicates ± standard deviation. Odor threshold range obtained from [35]. Different levels of significance (*p* < 0.01) are indicated with different letters.

Compound	Odour Threshold Range (mg/L) ^1^	Tea + SCOBY	Tea + SCOBY + *P. kluyveri*	Tea + *P. kluyveri*
Ethyl acetate (mg/L)	7.5–60	ND ^2^	11.2 ± 4.5	ND
Isoamyl acetate (µg/L)	0.25–1.8	ND	2136.7 ± 312.6	ND
2-Methylbutyl acetate (µg/L)	1.6	ND	254.8 ± 37.8	ND
2-Phenethyl acetate (µg/L)	0.03–1.8	12.4 ± 0.6 ^a^	567.5 ± 27.4 ^b^	71.6 ± 22.3 ^a^

^1^ based on [36], ^2^ ND—Not detected.

## Data Availability

Data is contained within the article.
